# Persona Development in Washington State: Mixed Methods Approach Using Statewide Survey Data

**DOI:** 10.2196/75422

**Published:** 2026-03-31

**Authors:** Jordan M Garcia, Rebecca A Hills, Debra Revere, Jaewon Lim, Adam S Elder, Chris Baumgartner, Bryant T Karras, Janet G Baseman

**Affiliations:** 1 School of Social Work University of Washington Seattle, WA United States; 2 Department of Epidemiology School of Public Health University of Washington Seattle, WA United States; 3 Department of Health Systems & Population Health School of Public Health University of Washington Seattle, WA United States; 4 Department of Biostatistics School of Public Health University of Washington Seattle, WA United States; 5 Washington State Department of Health Tumwater, WA United States

**Keywords:** personas, user-centered design, public health informatics, information system design, cluster analysis, qualitative data

## Abstract

**Background:**

Personas, fictional profiles representing user segments, play an important role in human-centered design, ensuring tools are tailored to the needs of users. Although public health organizations often develop information systems to promote population health, human-centered design methods and personas are generally underused in public health informatics projects.

**Objective:**

This study aims to present a novel, mixed methods approach to developing data-driven personas for use in public health information system design, leveraging 2 statewide surveys conducted in Washington State. The aim is to produce realistic, representative, and actionable personas that reflect the diversity of a state population and support user-centered design in public health initiatives.

**Methods:**

Quantitative (cluster analysis) and qualitative (thematic review and quote extraction) methods were applied to 2 statewide survey datasets: (1) a statewide knowledge, attitudes, and practices survey (N=1103) which used random, address-based sampling, and (2) a subset of the knowledge, attitudes, and practices respondents (N=143), which included more targeted questions on opinions and preferences related to public health information systems. Characteristics examined included demographics, technological readiness, opinions about public health policies, and experience using online health tools.

**Results:**

K-prototype clustering resulted in 5 clusters. These 5 clusters were studied using both quantitative and qualitative analysis of key factors of the Washington State population to build 13 personas. Each persona represents a different population demographic, varying levels of technological readiness and attitudes toward public health policies, and differing experiences with online health tools. Persona descriptions are further elucidated with a short profile and 2-3 quotes.

**Conclusions:**

This study offers a scalable and adaptable framework for persona development in public health, demonstrating how existing datasets can be transformed into effective design tools. Through a mixed methods approach, personas that reflect the diverse needs, preferences, and behaviors of Washington State residents were created. These personas can enhance the design, development, and evaluation of public health information systems by centering on user experience. Persona development and the methods described here can be used in future public health informatics projects to assist in formative research, guide design and development, inform usability testing, and shape communication strategies. By bridging the gap between large-scale data and user-centered design, this approach provides a practical model for making public health technologies more aligned with community needs.

## Introduction

Public health informatics projects face the challenge of designing tools that resonate with diverse populations while being applied universally [1,2]. Due to time pressures and limited resources of public health work, it is often difficult to gather sufficient user input to gain a nuanced understanding of the needs of the population being served. Further, surveys and population summaries may not adequately capture the complexity and variability in public attitudes and behaviors [3,4]. User-centered approaches can help bridge this gap by focusing on the specific needs of individuals [5]. One such user-centered design (UCD) tool, personas, can assist in representing individual needs [6]. This study focuses on the creation of detailed personas using a rich dataset, offering a novel approach to public health informatics design work that balances empirical data with granular insights.

Personas are fictional representations of individual users and may be best known for their use in marketing; however, personas are also used by informaticians, designers, and developers to better understand the needs, behaviors, and preferences of potential users of their products, systems, or services [7]. Generally, personas may include demographic information and behavioral patterns derived from user research. As part of a UCD approach, personas foster empathy with users, generating valuable insights for product design, development, and strategy [6]. Essentially, personas can provide a more concrete and actionable representation of both unstructured and complex user data, making it easier to interpret.

Although personas are widely used in information systems design, their application in health IT and public health informatics remains limited [8-11]. Public health efforts, in particular, could benefit from the use of personas, especially in contexts where resources to support direct user engagement and data collection are constrained. In public health, personas may be useful in various processes: the design and evaluation of products, recruiting for usability testing activities [12], and facilitating communication across stakeholder groups during development and decision-making processes [13]. In particular, personas can be an innovative strategy for public health teams by offering relatable, data-driven representations of target populations. This approach can enhance the reach and effectiveness of public health initiatives by keeping user needs at the forefront of design and implementation. Additionally, personas can support stronger communication and marketing efforts, making public health campaigns more engaging and accessible to diverse audiences. By integrating personas, teams may create more responsive, user-centered solutions that can improve public health outcomes.

Traditional methods of persona development often rely on small sample sizes or limited information, leading to less accurate and less representative user profiles [14]. Additionally, there is no standardized methodology, resulting in inconsistencies and differing priorities in the development process [15]. Some approaches prioritize rich qualitative data, obtaining the data from a very limited sample of the population. While these processes are designed to capture detailed individual experiences and perspectives, they may miss insights from unsampled portions of the population or key overarching trends [16]. In contrast, development processes focused on quantitative methods may use larger pools of user data, but generalizations may occur, and subtleties can be lost [17,18]. Mixed methods approaches seek to find a balance between these 2 [6]. This study emphasizes the importance of a comprehensive approach that integrates both robust quantitative metrics and detailed qualitative insights to create a more extensive and nuanced understanding of user experiences and needs.

The objective of this study is to develop a set of personas for use in public health informatics initiatives using a mixed methods approach. By synthesizing quantitative and qualitative data from representative datasets, this study aims to create realistic and representative personas that can inform the design and implementation of public health technologies. These personas are intended to support various project stages, including formative research, usability testing, and communication strategies, ensuring that public health technologies are tailored to meet the specific needs, preferences, and barriers faced by diverse population segments. This study demonstrates the efficacy of integrating quantitative and qualitative data in persona development, leveraging an approach that combines statistical rigor with contextual insights. The resulting approach provides a versatile template adaptable for other public health initiatives.

## Methods

### Context

WA Verify, developed by the Washington State Department of Health, is a smartphone-based tool launched in November 2021 that provides Washington residents with digital access to their COVID-19 vaccine records. To evaluate it, a statewide survey assessed knowledge, attitudes, and practices regarding public health technologies. Findings highlighted both high rates of internet and smartphone access and concerns about privacy and record reliability (Multimedia Appendix 1).

As pandemic restrictions eased, a second-phase survey was conducted in April 2023 to explore potential expanded uses for WA Verify and its underlying technology. This survey examined preferences and concerns related to privacy and security. Findings revealed that participants generally trusted public health tools but worried about the security of their personal health data. Nevertheless, many participants acknowledged the societal benefits of these tools, ultimately weighing the advantages over perceived risks.

The 2 rich datasets described above offered a unique opportunity to understand the Washington State population as a whole and capture some of the specific concerns and opinions of Washington residents in their own words. In public health, time and resource limitations may hinder optimal user input during the planning and design of informatics projects. To address this gap, personas emerged as 1 way to leverage the available data in order to represent users and succinctly communicate their needs and preferences. A persona is a fictitious individual created to represent a user group, providing designers with personal stories that offer insights into motivations and behaviors. While traditional UCD efforts often rely on small sample sizes and qualitative data, the 2 evaluation survey datasets with rich representative data, as well as focused qualitative data on informatics topics, presented an opportunity to create a set of data-derived personas. The goal was to design personas that exhibit the following attributes:

1. Simple and relatable: provide a collection of distinct individuals who can be understood at an intuitive and personal level to help with product design.
2. Accurate: as much as possible, accurately represent the relationship between demographic characteristics and technology-related tendencies.
3. Diverse: represent a range of characteristics. While not capturing every individual, the set of personas should encapsulate diverse attributes, encouraging consideration of various population segments.
4. Actionable: provide insights that can inform decisions and strategies in public health informatics projects.
5. Comprehensive: elucidate motivations, intentions, and needs, not just demographics.

In the development process, these persona attributes may be at odds with one another. For example, the goals of relatability and actionability may conflict with complete statistical accuracy. This approach seeks to find a balance among these goals in order to create a data-driven and useful set of personas.

### Data Collection

The WA Verify evaluation project conducted 1 statewide survey and later a second-phase survey; data from these 2 surveys were used for persona development. Survey methods are summarized below and are described in detail elsewhere [19].

The initial statewide survey was mailed to a random sample of 5000 Washington households between September 2022 and September 2023, with 1491 responses (32% response rate). It assessed technology use, digital literacy, internet access, experiences with COVID-19 verification, and attitudes toward public health tools.

The second-phase survey targeted a smaller sample but aimed to gather more specific information about barriers to technology use in the public health context. The survey focused on digital literacy, internet safety perceptions, digital privacy and security, confidence in public health initiatives, and opinions on hypothetical public health tools, including some space for free responses from participants to elaborate on their views. The complete second-phase survey instrument is included in Multimedia Appendix 2. The survey sample consisted of 321 respondents from the statewide survey who expressed interest in future studies, approximately 20% of all initial respondents. Data collection took place in April 2023, yielding responses from 143 individuals (44.5% response rate).

### Data Analysis

**Figure 1:**
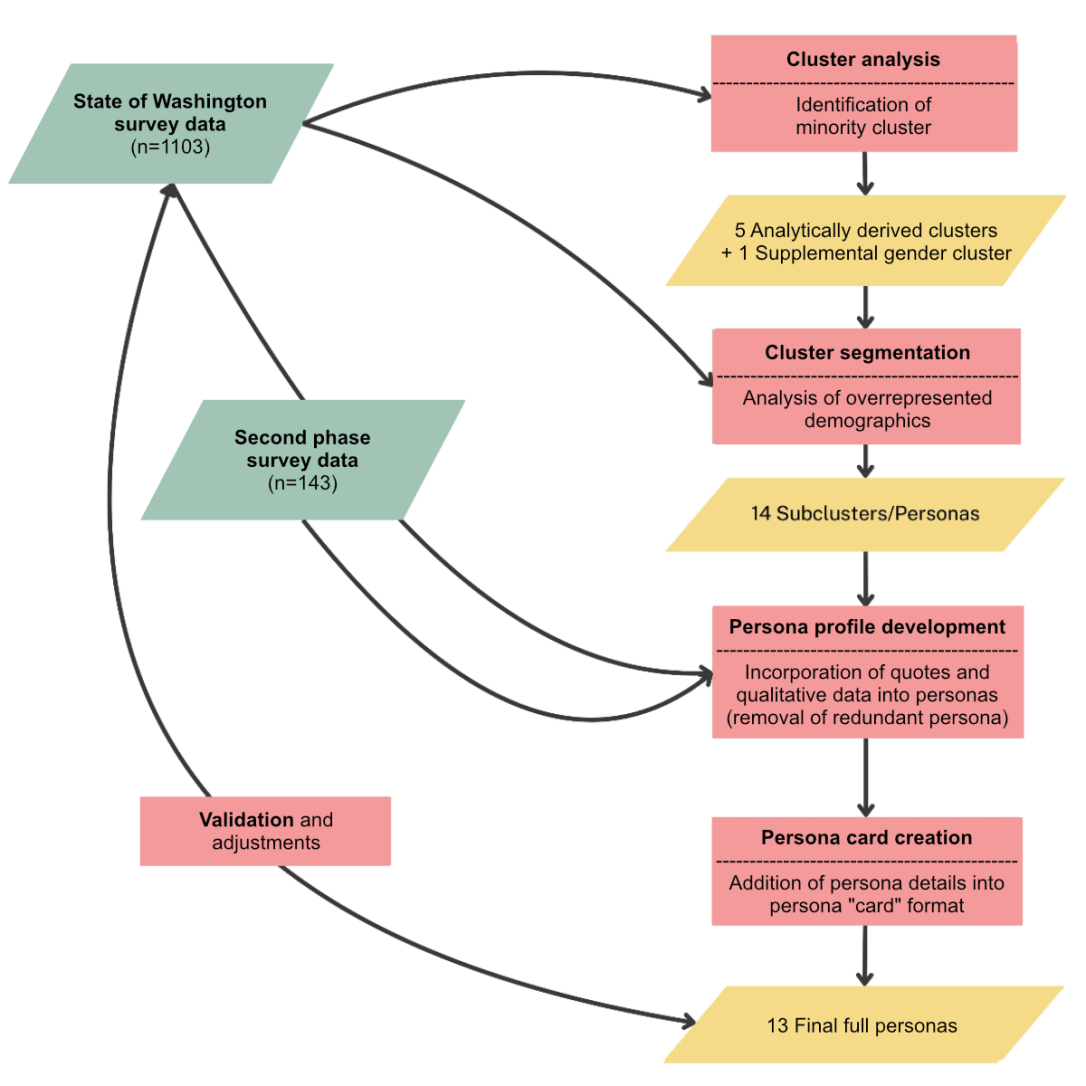
Steps of the persona development process.

Personas were developed from the 2 survey datasets using a mixed methods approach, combining quantitative and qualitative analyses. While cluster analysis provided a statistical foundation, ensuring that the final product was aligned with the survey data, this method alone did not allow for comprehensive personas. The mixed methods approach allowed for refinement of the initial clusters into distinct groups with specific demographic characteristics, aiming to capture the specific needs of diverse user groups. The process involved 5 main steps: cluster analysis, cluster segmentation, persona profile development, persona card creation, and validation. Figure 1 provides a graphical representation of this process, and each step is summarized below.

1. Cluster analysis: cluster analysis identified 5 primary clusters based on 14 key variables, including demographic, technological, and policy opinion characteristics from the State of Washington survey data. An additional cluster was created to represent nonbinary and transgender individuals who did not have sufficient representation to be included in the primary cluster analysis.
2. Cluster segmentation: quantitative methods expanded the 5 primary clusters into 14 subclusters or cluster segments. Joint distributions of age, sex, and race and ethnicity were examined to create distinct cluster segments within each cluster. Outcome variables were then summarized and assigned to these segments, creating personas. Race and ethnicity were combined into a single variable, which categorized individuals into one of five groups. All individuals who indicated they were Hispanic were categorized as “Hispanic.” Among those who did not indicate they were Hispanic, individuals who indicated they were only White, only Black, or only Asian were categorized as “White,” “Black,” and “Asian,” respectively. All other individuals (including individuals who selected multiple races) were categorized as “Other.”
3. Persona profile development: qualitative data integration enriched personas with quotes and themes extracted from both survey datasets. This process added depth and nuance to personas with additional characteristics and narrative descriptions. Redundant personas were consolidated, resulting in a final set of 13 personas.
4. Persona card creation: after defining the personas, persona cards were developed to visually represent each persona’s characteristics, needs, and motivations.
5. Validation: a validation process ensured that the personas accurately reflected the demographic and attitudinal diversity observed in the Washington datasets. Team review and quantitative comparison methods were used to iteratively refine and validate each persona.

### Quantitative Methods

#### Cluster Analysis

To identify distinct groups within the dataset, K-prototype clustering with Gower’s distances [20] was used. K-prototype clustering is an extension of K-means that is suitable for datasets with numerical and categorical variables [21,22]. A distance function was defined to create clusters based on variables of interest, producing a “distance” value indicating similarity between respondents, with smaller values denoting greater similarity and larger values less similarity. The 14 variables used in this analysis can be seen in Table 1.

**Table 1:** Cluster analysis variables.

Variables			Description		Cluster analysis categories
**Demographics**					
	Age (years)	What is your age?		18-39; 40-59; 60+	
	Sex	Are you _________?		Female; male; transgender; nonbinary and nonconforming; Prefer not to respond	
	Race and ethnicity	How would you describe your race and ethnicity?		Non-Hispanic White (White); Non-Hispanic Black (Black); Hispanic any race (Hispanic); Non-Hispanic Asian (Asian); Non-Hispanic of another race or ethnicity (Other)	
	Geographic region	Federal Information Processing Standard Code		Eastern Washington; Western Washington	
	English only at home	Do you speak a language other than English at home?		Only English; language other than English	
	Parental status	Are you a parent or guardian to a child under 18 years old?		Parent or guardian to <18-year-old; Not a parent or guardian to <18-year-old	
	Education	What is your highest level of education?		No high school degree; high school graduate; Some college or 2-year degree; 4-year degree or more	
**Technological and nondemographic variables**					
	Technological readiness	Calculated variable: overall, how confident, if at all, do you feel using computers, smartphones, or other electronic devices to do the things you need to do online? How true is the following statement? “When I get a new electronic device, or need to do a new task on it, I usually need someone else to set it up, show me how to use it, or help me.”		Higher technological readiness; lower technological readiness	
	eHealth tool usage	Have you ever used any electronic health tools?		Used eHealth tool; did not use eHealth tool	
	Method of learning of WA Verify	How did you first hear about WA Verify?		Health care; never heard; news; other; referral	
	WA Verify usage	Calculated Variable: Do you use WA Verify? How willing would you be to use a portable electronic COVID-19 vaccine record?		Yes; willing; not willing	
	COVID policy opinion	How do you feel about policies that require proof of vaccination or a negative COVID-19 test result to enter spaces that are high risk of COVID-19 spread?		Support; oppose	
	Health portal usage	Have you ever used any electronic health tools?: An online patient medical record or health portal (eg, MyChart).		Yes; no	
	WA Notify usage	Have you ever used any electronic health tools?: WA Notify (WA's COVID-19 Exposure Notification Tool).		Yes; no	
	Tracking apps usage (tracking)	Have you ever used any electronic health tools?: A tool for keeping track of my activities like diet and exercise (eg, Fitbit, Strava, or MyFitnessPal)		Yes; no	

Since the personas were specifically developed in the context of informing the design and implementation of digital public health tools, it was crucial to differentiate between groups based on technology-related factors. To ensure that the cluster reflected a range in technology adoption, technology-related variables were weighted more heavily (5:1 relative to demographic variables). Exploratory testing with alternative weights (eg, 3 and 10) yielded similar results, with 5:1 providing a balanced emphasis on them.

Construction of the clusters was carried out by maximizing the 50-fold cross-validated silhouette score [23]. The silhouette score, ranging from –1 to 1, measures how similar each data point is to its own cluster compared to other clusters, with higher scores indicating better-defined clusters. This approach enhances the reliability and accuracy of the results by allowing for the assessment of clustering performance on data not used in its construction, reducing overfitting risk, improving cluster stability, and enhancing generalizability across different data subsets. The optimal number of clusters was determined to be 5, balancing granularity and meaningful segmentation within the dataset.

#### Addition of a Supplemental Cluster

Special methods were used to ensure the inclusion of demographic groups with limited presence in the sample, particularly nonbinary and transgender individuals. Due to the small number of survey respondents identifying as nonbinary or transgender, the primary cluster analysis focused exclusively on respondents identifying as male or female. To address this limitation and ensure representation of the gender diversity present in Washington State, an additional, supplemental cluster (supplemental gender cluster) was created, including only respondents who identified as nonbinary or transgender. The cluster’s summary characteristics were defined using an aggregate of the characteristics from the 8 respondents who comprised this group.

#### Cluster Segmentation

Because the initial clusters still contained substantial diversity, they were further segmented by age, race and ethnicity, and sex, which are factors that are known to influence behaviors and attitudes toward health tools [24-29]. Overrepresented demographics within clusters were split into additional subgroups, yielding 13 personas.

An example of the segmentation process can be seen in Table 2, which compares the distribution of the entire sample to that of Cluster 1. Cluster 1 had a higher proportion of White, male, 40- to 59-year-olds as well as Asian, female, 40-59-years prompting division into 2 subclusters, each representing one of these identified groups (White, male, 40-59 years and Asian, female, 40-59 years).

**Table 2:** Three-way table of age, sex, and race and ethnicity among the total survey sample (n=1103) and Cluster 1 (n=272) with observed/expected for cluster categories.

Age and sex				All				White					Black				Hispanic				Asian				Other		
				Survey, n		Cluster 1 (O/E), n (%)		Survey, n			Cluster 1 (O/E), n (%)		Survey, n		Cluster 1 (O/E), n (%)		Survey, n		Cluster 1 (O/E), n (%)		Survey, n		Cluster 1 (O/E), n (%)		Survey, n		Cluster 1 (O/E), n (%)
**18-39** **years**																											
		Female	182		46 (102)		106			27 (103)		8		2 (101)		25		5 (81)		18		6 (97)		25		6 (97)	
		Male	110		22 (81)		67			12 (73)		2		0 (0)		6		2 (135)		19		5 (107)		16		3 (76)	
**40-59** **years**																											
		Female	208		74 (144)				155	53 (139)		5		0 (0)		16		4 (101)		19		11 (235)^a^		13		6 (187)	
		Male	136		59 (176)				106	49 (187)^a^		5		1 (81)		2		1 (203)		11		4 (147)		12		4 (135)	
**60+** **years**																											
		Female	259		43 (67)		225			38 (68)		1		0 (0)		7		0 (0)		4		1 (101)		22		4 (74)	
		Male	208		28 (55)		173			22 (52)		7		0 (0)		1		0 (0)		11		3 (111)		16		3 (76)	

^a^Results were larger than expected if clusters were assigned at random across race and age.

This segmentation process, applied to each of the 5 quantitatively derived clusters, resulted in 13 distinct personas. When combined with the Supplemental Gender Cluster, which remained a standalone persona without further subdivision, a total of 14 personas were created. Outcome variables of interest (eg, technological readiness, WA Verify usage, etc.) were identified for each persona. Most subclusters were defined by the demographic characteristics determined during cluster segmentation and the most frequently observed values of the outcome variables of interest within that subcluster.

Additionally, some personas were modified to better capture the diversity of demographics and outcome variables in the population. The distribution of variables in subclusters was compared to the overall statewide population to ensure representativeness. Since each persona can only reflect 1 value per variable, it is challenging to establish actionable, diverse, and representative personas. To address this, less common subcluster characteristics were sometimes selected over more common characteristics to improve diversity and representation. Further modifications are detailed in the Validation section, ensuring that the personas are data-driven and representative of Washington State's population.

### Qualitative Methods

#### Analysis Goals

Qualitative analysis added depth to the personas by incorporating participant quotes and themes to capture user perspectives, needs, and experiences. Thematic analysis identified recurring themes across responses, revealing common sentiments. This process added qualitative depth and real-world context to each persona.

#### Persona Profile Development

Personas were then synthesized and refined to ensure realistic, data-grounded representations of individuals. Key aspects crucial for constructing comprehensive personas [30] were identified and aligned with best practices for persona development [31,32], then tailored to incorporate information specifically relevant to public health information system efforts. These aspects included pertinent demographic information, public health opinions, quotes, and drivers (including needs and motivations).

Persona development and refinement took place as an iterative process with 3 University of Washington (UW) team members reviewing personas, examining quantitative and qualitative data, and reviewing and revisiting personas. The following additions and adjustments were made to personas during the iterative refinement process (not necessarily in this order):

1. Additional stories were built around the personas based on qualitative survey data from individuals with matching or similar characteristics to the persona.
2. Personas were given exact ages, marital statuses, and occupations;
3. Quotes and variations on quotes were assigned to personas from survey respondents with similar characteristics.
4. New quotes were created where only partial ideas existed in the qualitative data.
5. Personas were given names and pictures.
6. One persona was removed (cluster 2, persona 7) as seen in
  Table 3
  .

**Table 3:** Primary persona list derived from expansion of cluster analysis groupings.

Persona number	Cluster number	Age group (y)	Sex	Race or ethnicity	Region	English only	Parent	Education	Tech readiness	WA Verify refer	WA Verify usage	Opinion COVID policies	Health portal	WA Notify usage	Tracking
1	1	40-59	Male	White	W^a^	Yes	No	4-year	High	Referral	Yes	Support	Yes	Yes	Yes
2	1	40-59	Female	Asian	W	No	Yes	4-year	High	Referral	Yes	Support	Yes	Yes	Yes
3	2	18-39	Female	White	W	Yes	No	4-year	High	Referral	Willing	Support	Yes	Yes	Yes
4	2	18-39	Male	White	W	Yes	No	4-year	High	Never heard	Willing	Support	Yes	Yes	Yes
5	2	18-39	Male	Black	W	Yes	No	4-year	High	Never heard	Willing	Support	Yes	Yes	Yes
6	2	18-39	Female	Hispanic	W	Yes	No	≤2-year	High	Never heard	Willing	Support	Yes	Yes	Yes
7	2	18-39	Male	Asian	W	Yes	No	4-year	High	Never heard	Willing	Support	Yes	Yes	Yes
8	3	60+	Female	White	W	Yes	No	≤2-year	Lower	Never heard	Not Willing	Support	Yes	No	No
9	3	60+	Male	White	W	Yes	No	≤2-year	Lower	Never heard	Willing	Support	Yes	No	No
10	4	60+	Female	White	W	Yes	No	4-year	Lower	News	Willing	Support	Yes	Yes	Yes
11	5	18-39	Male	White	W	Yes	No	4-year	High	Never heard	Willing	Oppose	Yes	Yes	Yes
12	5	40-59	Female	Black	W	Yes	No	4-year	High	Referral	Willing	Support	Yes	No	Yes
13	5	18-39	Female	Hispanic	E^b^	No	Yes	≤2-year	High	Never heard	Willing	Oppose	Yes	No	Yes
14	Supp.	18-39	Nonbinary, Transgender	White	W	Yes	No	4-year	High	Never heard	Willing	Support	Yes	Yes	Yes

^a^W: Western Washington.

^b^E: Eastern Washington.

During the refinement process, representative quotes and key motivations (drivers) were added to each persona. Quotes were selected during the qualitative analysis and matched to demographically similar personas, with minor adjustments for clarity and brevity. Drivers emerged from thematic analysis and were grounded in survey data. Additionally, as mentioned above, 1 persona was removed because it reflected too small a respondent group and overlapped considerably with others from the same cluster. In line with our refinement criteria, we kept only personas that were both supported by sufficient data and represented distinct needs and motivations. Throughout this process, the balance between sufficient detail and making realistic personas while avoiding stereotypes and oversimplification was carefully considered.

#### Persona Card Creation

Following the development of the demographic profiles, persona cards were created to serve as visual and textual representations of the personas. The information about each persona derived from the steps previously outlined was translated into a concise format to facilitate their use in design and decision-making processes. First, a standard template was identified to outline the sections for demographics, public health opinions, quotes, and drivers. The persona demographic profiles were transferred into this template. Each persona card also included a representative image, which was selected to be demographically appropriate and aligned with the persona’s profile. After the card drafts were established, they were reviewed by UW team members, looking for redundancies and inconsistencies. This process resulted in 13 persona cards.

#### Validation

Because the process by which the personas were developed was novel, and in some cases ad hoc, a validation step was used to confirm that the final persona characteristics were reflective of the observed data. Each persona was validated using the following steps:

1. A group of respondents was created for each persona where each record matched the following characteristics exactly: age group, sex, race and ethnicity, geographic region, language at home, parental status, and level of education.
2. In some cases, when this group of respondents was small or non-existent, a second, larger subset was also considered that included both exact matches and individuals that matched on all but 1 of the 7 characteristics mentioned in Step 1.
3. The distributions of technological readiness, opinions on COVID-19 policies, use of digital health portals, and use of health tracking software were tabulated and compared to the characteristics assigned to the given persona.
4. In cases where a mismatch between the persona characteristic and the respondent summary was observed, the personas team discussed and came to a collaborative decision on whether to make a change to the persona characteristic.

In total, 4 major discrepancies were identified through this process, and each prompted a discussion among the team. In 2 cases, the group determined the persona’s characteristics should reflect the demographic groupings, and changes were made to reflect this. However, in 2 cases, the discrepancy was retained. For both affected personas, a thoughtful decision was made to retain groups who are generally less represented, likely have unique needs, and are not well understood with respect to habits and preferences regarding public health information systems (ie, low technological readiness and less experience with online health tools). These decisions reflect the belief that although some characteristics are less common in the generally tech-savvy population of Washington State, it is important to take less common needs and traits into consideration to design accessible tools for all Washington residents.

### Ethical Considerations

In June 2022, the UW Human Subjects Division reviewed the WA Verify evaluation project (IRB ID: STUDY00015786) and determined it did not qualify as research under federal and state regulations, thus exempting it from UW Institutional Review Board review. The Washington State University/Social and Economic Sciences Research Center Institutional Review Board subsequently confirmed this exempt status. Survey participants were asked to confirm their agreement to participate in the survey before starting the survey. No secondary data was used in this analysis.

## Results

### Overview

Overall, 13 personas emerged through the development process, 12 derived from the quantitative clustering and expansion process, and 1 Supplemental Gender Cluster.

### Clusters

Cluster analysis identified 5 clusters. Additionally, a supplemental cluster was defined to identify a segment of the survey population not considered in the analysis. Table 4 displays these 6 clusters. Additional summary tables of the sample for related questions from the survey are provided in Multimedia Appendix 3.

**Table 4:** Five quantitatively identified clusters and one supplemental gender cluster with summary characteristics.

Characteristics			All (N=1103)		Cluster 1 (n=272)		Cluster 2 (n=223)		Cluster 3 (n=302)		Cluster 4 (n=57)		Cluster 5 (n=249)		Supplemental (n=8)
**Age (years), n (%)**															
	18-39	292 (26)		68 (25)		97 (43)^a^		7 (2.3)		0 (0)		120 (48)^a^		5 (63)^a^	
	40-59	344 (31)		133 (49)^a^		68 (30)		36 (12)		17 (30)		90 (36)		1 (13)	
	60+	467 (42)		71 (26)		58 (26)		259 (86)^a^		40 (70)^a^		39 (16)		2 (25)	
**Sex, n (%)**															
	Female	649 (59)		163 (60)		128 (57)		164 (54)		44 (77)^a^		150 (60)		0 (0)	
	Male	454 (41)		109 (40)		95 (43)		138 (46)^a^		13 (23)		99 (40)		0 (0)	
	Nonbinary	0 (0)		0 (0)		0 (0)		0 (0)		0 (0)		0 (0)		5 (63)	
	Transgender	0 (0)		0 (0)		0 (0)		0 (0)		0 (0)		0 (0)		3 (38)	
**Race and ethnicity, n (%)**															
	White	832 (75)		201 (74)		156 (70)		251 (83)^a^		48 (84)^a^		176 (71)		5 (63)	
	Black	28 (2.5)		3 (1.1)		8 (3.6)^a^		5 (1.7)		1 (1.8)		11 (4.4)^a^		0 (0)	
	Hispanic	57 (5.2)		12 (4.4)		18 (8.1)^a^		8 (2.6)		0 (0)		19 (7.6)^a^		1 (13)	
	Asian	82 (7.4)		30 (11)^a^		21 (9.4)^a^		13 (4.3)		4 (7)		14 (5.6)		1 (13)	
	Others	104 (9.4)		26 (9.6)		20 (9)		25 (8.3)		4 (7)		29 (12)		1 (13)	
**Region, n (%)**															
	Eastern	205 (19)		37 (14)		35 (16)		69 (23)^a^		9 (16)		55 (22)^a^		1 (13)	
	Western	898 (81)		235 (86)^a^		188 (84)^a^		233 (77)		48 (84)^a^		194 (78)		7 (88)^a^	
**English, n (%) **															
	Only English	969 (88)		230 (85)		191 (86)		281 (93)^a^		53 (93)^a^		214 (86)		8 (100)^a^	
	Other language	134 (12)		42 (15)		32 (14)		21 (7)		4 (7)		35 (14)		0 (0)	
**Parent to child <18, n (%)**															
	Yes	271 (25)		83 (31)^a^		60 (27)		23 (7.6)		7 (12)		98 (39)^a^		0 (0)	
	No	832 (75)		189 (69)		163 (73)		279 (92)^a^		50 (88)^a^		151 (61)		8 (100)^a^	
**Education, n (%) **															
	4-year degree or more	657 (60)		207 (76)^a^		146 (65)^a^		123 (41)		38 (67)^a^		143 (57)		6 (75)	
	2-year or some college	290 (26)		53 (19)		58 (26)		101 (33)^a^		15 (26)		63 (25)		1 (13)^a^	
	High school graduate	123 (11)		11 (4)		18 (8.1)		58 (19)		4 (7)		32 (13)^a^		1 (13)^a^	
	Less than high school	33 (3)		1 (0.4)		1 (0.4)		20 (6.6)		0 (0)		11 (4.4)^a^		0 (0)^a^	
**Technological readiness, n (%)**															
	Higher	826 (75)		256 (94)^a^		219 (98)^a^		97 (32)		13 (23)		241 (97)^a^		8 (100)^a^	
	Lower	277 (25)		15 (5.9)		4 (1.8)		205 (68)^a^		44 (77)^a^		8 (3.2)		0 (0)	
**Method of learning of WA Verify, n (%)**															
	Referral	223 (20)		136 (50)^a^		26 (12)		17 (5.6)		10 (18)		34 (14)		1 (13)	
	News	144 (13)		53 (19)^a^		11 (4.9)		31 (10)		27 (47)^a^		22 (8.8)		0 (0)	
	Health care	117 (11)		62 (23)^a^		19 (8.5)		19 (6.3)		3 (5.3)		14 (5.6)		0 (0)	
	Others	47 (4.3)		17 (6.3)		11 (4.9)		8 (2.6)		3 (5.3)		8 (3.2)		1 (13)	
	Never heard	572 (52)		4 (1.5)		156 (70)^a^		227 (75)^a^		14 (25)		171 (69)^a^		6 (75)^a^	
**WA Verify usage, n (%)**															
	Yes	276 (25)		234 (86)^a^		5 (2.2)		21 (7)		7 (12)		9 (3.6)		1 (13)	
	Willing	560 (51)		17 (6.3)		193 (87)^a^		150 (50)		38 (67)^a^		162 (65)^a^		6 (75)^a^	
	Not willing	267 (24)		21 (7.7)		25 (11)		131 (43)		12 (21)		78 (31)^a^		1 (13)	
**COVID policies, n (%)**															
	Oppose	226 (20)		26 (9.6)		27 (12)		69 (23)^a^		8 (14)		96 (39)^a^		0 (0)	
	Support	877 (80)		246 (90)^a^		196 (88)^a^		233 (77)		49 (86)^a^		153 (61)		8 (100)^a^	
Health portal, n (%)			949 (86)		262 (96)^a^		212 (95)^a^		199 (66)^a^		57 (100)^a^		219 (88)^a^		7 (88)^a^
WA Notify usage, n (%)			535 (49)		235 (86)^a^		223 (100)^a^		28 (9.3)^a^		49 (86)^a^		0 (0)		2 (29)^a^
Tracking, n (%)			660 (60)		209 (77)^a^		181 (81)^a^		24 (7.9)^a^		45 (79)^a^		201 (81)^a^		6 (75)^a^

^a^Results were larger than expected based on the full sample.

### Persona Segments

From the 5 calculated clusters and the supplemental gender cluster, 14 personas were identified to represent distinct segments of the population based on race and ethnicity, age, and sex. By identifying overrepresentation of these demographic characteristics, personas were defined within each cluster. This expanded cluster-derived persona list is displayed in Table 3.

### Persona Profiles

To enhance the initial persona profiles derived from cluster analysis and segmentation, qualitative analysis was conducted. This process incorporated demographic data and qualitative responses from both surveys to identify the needs, preferences, and experiences of each persona. This approach allowed us to humanize the personas. Tables 5 and 6 present the additional attributes developed through this qualitative analysis, providing a richer, more nuanced description of each persona.

**Table 5:** Final persona list with demographic information.

Persona number	Cluster number	Age (years)	Sex	Race and ethnicity	Region	English only	Parent	Education	Tech readiness
1	1	40-59	Male	White	Western	Yes	Yes	4-year	High
2	1	40-59	Female	Asian	Western	No	Yes	4-year	High
3	2	18-39	Female	White	Western	Yes	No	4-year	High
4	2	40-59	Male	White	Western	Yes	No	4-year	Lower
5	2	18-39	Male	Black	Western	Yes	No	4-year	High
6	2	18-39	Female	Hispanic	Eastern	Yes	No	≤ 2-year	High
7	3	60+	Female	White	Western	Yes	No	≤ 2-year	Lower
8	3	60+	Male	White	Western	Yes	No	≤ 2-year	Lower
9	4	60+	Female	White	Western	Yes	No	4-year	High
10	5	18-39	Male	White	Eastern	Yes	Yes	4-year	High
11	5	40-59	Female	Black	Western	Yes	Yes	4-year	High
12	5	18-39	Female	Hispanic	Western	No	No	≤ 2-year	High
13	Supp.	18-39	Nonbinary	White	Western	Yes	No	4-year	High

**Table 6:** Final persona list with profiles and names.

Persona number	Cluster number	Name	Age (years)	Marital status	Occupation
1	1	David	43	Divorced	Software Engineer
2	1	Yuki	40	Married	PT Assistant
3	2	Emily	32	Single	Social Services
4	2	Ryan	52	Single	Grocery Stocker
5	2	Desmond	37	Married	Banking
6	2	Maria	35	Married	Human Resources
7	3	Linda	66	Married	Retired
8	3	Richard	60	Divorced	Appliance Repair
9	4	Patricia	67	Married	Retired
10	5	Kyle	31	Married	Firefighter
11	5	Alicia	46	Re-married	Teacher
12	5	Isabel	21	Single	Premedical student
13	Supplemental	Riley	26	In a relationship	Graduate Student

### Persona Cards

**Figure 2:**
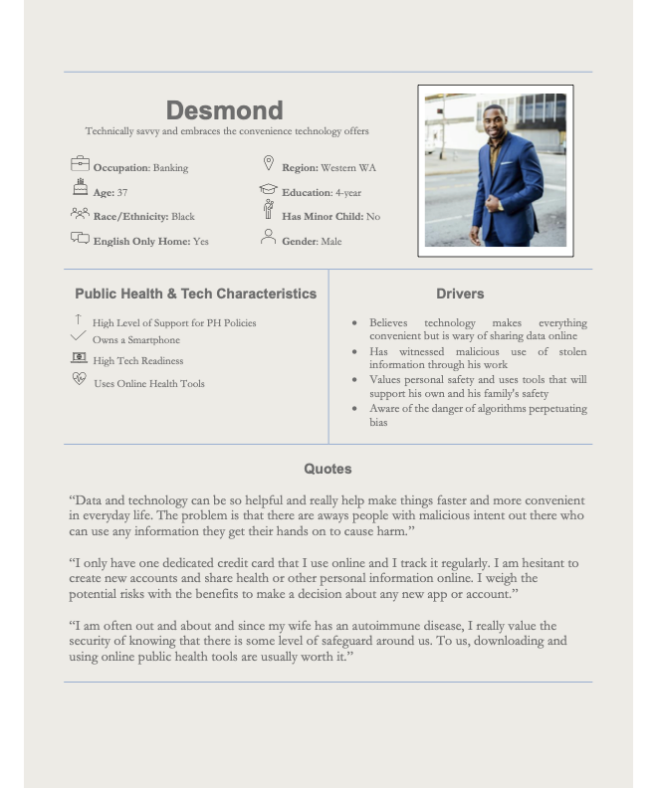
Persona 5: Desmond.

The final 13 personas were formatted into graphic single-page summaries or persona cards. Figure 2 depicts 1 persona card (Personas 5 from Table 5). Quotes and drivers were also added to the personas at this stage.

**Figure 3:**
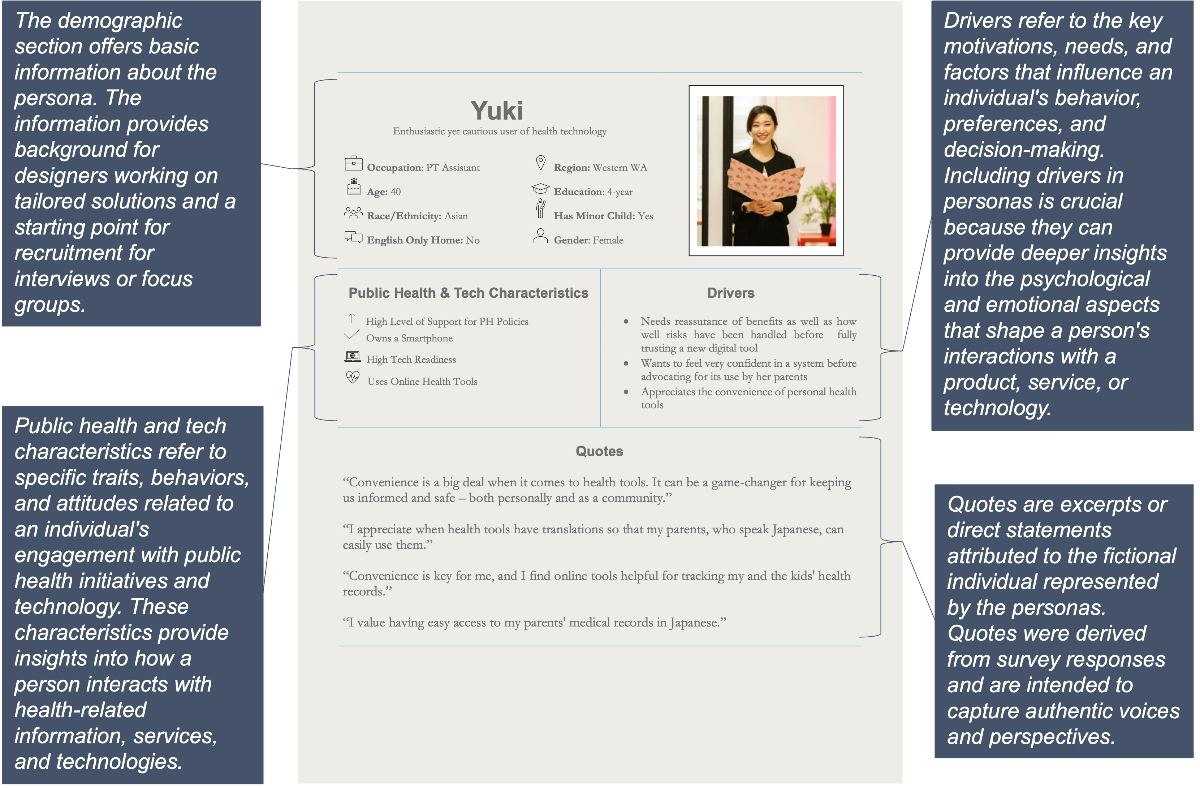
Sample persona with section descriptions.

Aspects of the persona cards were included based on their ability to provide a practical and user-friendly tool to bridge the gap between data and actionable insights. A detailed description of the derivation and relevance of each section is provided in Figure 3.

The full set of 13 persona cards can be found in Multimedia Appendix 4 and is available for download, allowing researchers and practitioners to use and adapt them for their own work.

## Discussion

### Principal Findings

The development of these personas leveraged a uniquely rich dataset, enabling a nuanced representation of diverse population segments. The mixed methods approach combined a rigorous data-driven process with the depth of individual stories, behaviors, and needs. This synthesis achieved a deeper understanding of the dataset and allows for quickly gleanable insights, balancing the key priorities for the persona creation, which could not be accomplished with quantitative or qualitative methods alone.

The approach focused on creating personas that were simple and relatable, accurate, diverse, actionable, and comprehensive. At times, decisions in the methodology had to be made to ensure effectiveness by finding a balance among these guiding principles. The aim was to define a collection of distinct individuals who could be intuitively understood. The personas reflect the breadth of demographic diversity in Washington State, representing a range of characteristics rather than matching exact population proportions, ensuring actionable insights for public health informatics projects.

Cluster analysis served as a starting point, ensuring the final personas were guided by the survey data. While cluster analysis alone was the most statistically accurate process, these clusters did not establish a set of personas that upheld the other key priorities. Through a mixed methods approach, distinct groups with more specific demographic characteristics were constructed. This technique helps highlight various user groups who might have more specific needs. For example, some of the personas can help understand challenges for those who have lower technological readiness that could benefit from targeted strategies regarding technology adoption and usage. While this is a minority of the population, individuals with these needs must still be considered in design, development, and communication.

This methodology aimed to mitigate potential biases from both human-driven and purely quantitative approaches to persona development. Traditional persona creation often relies heavily on individual interpretation, which can introduce unconscious human biases and assumptions. Conversely, purely data-driven approaches can amplify existing biases in the source data, which can result in further marginalizing groups underrepresented in the dataset. The mixed methods approach used in this study leverages the strengths of both while working to mitigate their weaknesses. The cluster analysis provided a foundation that reduced subjective biases in the initial grouping, while the qualitative analysis preserved a human perspective, allowing for the creation of authentic and relatable personas that addressed gaps in the quantitative results. This approach ensures statistically grounded and representative personas.

This development process considered a wide range of demographic and outcome variables, ensuring the inclusion of diverse population segments. This comprehensive approach is crucial in public health initiatives, where efforts impact a broad population in highly personal and individual ways. While resource-intensive, this approach offers a useful framework for creating representative and realistic personas. The template may be used and adapted to meet the specific needs of various projects.

This methodology demonstrates the value of a mixed methods approach in capturing the complexity of public health user groups and provides an adaptable template for future initiatives.

### Use of Personas in Public Health Informatics Projects

The personas developed through this process may serve as a tool to inform and guide public health informatics initiatives in the following ways:

1. Facilitate formative work: prior to embarking on a development project, personas can be used to understand potential users and recruit for interviews and focus groups, during which tailored information needs and user requirements data can be collected and analyzed.
2. Design and development: the design and development process can be aided by personas, providing an understanding of user preferences and behaviors. Decision-making can be aided by considering the impact of a tool or change on all target personas.
3. Usability testing: personas can be used to either simulate user interactions during usability testing or assist in the recruitment of diverse and appropriate usability testers.
4. Communications: personas can be used to facilitate internal communications (ie, development team decision-making) and external communications (eg, deciding on targets and strategies for education and information campaigns).

These personas can be used directly in practice. For example, a campaign focused on changing the health behaviors of Washingtonians could use these personas to consider how to tailor information to individuals with varying priorities and levels of digital comfort. Application of these personas can bring in considerations of real user opinions.

### Implications for Public Health Initiatives

Understanding the needs and motivations behind different personas can enhance the effectiveness of public health interventions. Strategies can be tailored to specific personas, and more targeted and impactful campaigns and technologies can be implemented. A user-centered design process that is still based on comprehensive data enhances usability and ensures that interventions resonate with distinct segments of the population. This may result in higher engagement and better outcomes.

Further, as new data become available or as public health priorities shift, personas can be updated or added to reflect these changes, ensuring that the technology remains relevant and responsive to current needs. This adaptability is particularly valuable in the rapidly evolving landscape of public health, where timely and accurate information is crucial.

Moreover, the iterative and flexible nature of persona development allows for continuous refinement and adaptation. The personas developed in this study can serve as a template that other public health initiatives can modify according to their specific needs and available resources. Even without access to extensive datasets, personas can be developed using smaller datasets, targeted surveys, focus groups, or stakeholder interviews. This process and the final product can be adapted to whatever resources are available and relevant to the effort. This adaptive approach ensures that initiatives remain timely and responsive and allows for a meaningful tool across varying resource availability and project scales.

### Limitations

There are several important limitations to consider regarding both the development and potential use of these personas. Most importantly, while personas are valuable tools for design and development, they are not substitutes for direct user engagement and feedback. These personas should ideally be used in conjunction with, rather than in place of, real user data and testing. They are meant to serve as practical tools when user engagement is challenging or impossible due to resource constraints, but they should not be seen as comprehensive replacements for actual user research.

Additional limitations stem from the survey data on which these personas are based. First, relying on survey data introduces sampling bias, particularly in the case of the second-phase survey data, as survey respondents may not be representative of the diverse population of Washington State. Second, survey data were collected in the context of the COVID-19 pandemic, a moment in time. Public opinions rapidly evolve, and the sentiments expressed in the surveys and subsequently used to develop the personas may only reflect that context and not be applicable to a new context. In particular, survey participation during the pandemic may have been lower among certain groups, such as lower-income populations [33], and public attitudes at the time reflected heightened concern about health and strong adherence to public health guidelines compared to non-pandemic periods [34]. These factors may limit the generalizability of the personas to future public health informatics topics. Third, the surveys focused on capturing opinions regarding WA Verify and its potential expanded use cases, so generalizing to other public health tools will depend on each tool and its context.

The methodology used presents additional limitations. Cluster analysis requires incorporating assumptions and parameter choices that may impact their interpretation, reliability, and generalization outside the context of the analysis. The development of personas may inherently oversimplify the complexity of each individual represented. In addition, bias may be introduced into the personas by including demographic and other characteristics in their development.

The addition of the supplemental gender cluster, while intended to include individuals who identify as a minority gender group, presents another limitation. Personas are not meant to be statistically representative of the population, but their value is in simplifying a complex population into a small set of fictional individuals that can be connected with. However, this simplification comes with the cost of not capturing the breadth of experiences across the population. While the representation of gender-diverse individuals in this study does not fully capture the range of identities in Washington State and oversimplifies, it offers an important point of visibility and inclusion, ensuring these perspectives are considered within the broader set of personas.

### Future Work

The overall usefulness of these personas would benefit from an iterative process of feedback with stakeholders, including developers, designers, communication teams, and any other potential users of the personas. Stakeholders who are experienced with using personas, as well as those new to using personas, would provide valuable feedback. This process can help to ensure the relevance and applicability of these tools.

In particular, 1 question to be addressed is the appropriate level of detail that is useful for these personas. In UCD, there are differing opinions on the optimal level of detail in personas. Emerging ways of thinking promote more pared-down personas where there is no image and fewer personal details, focusing instead on only the practical elements that directly affect usability [35,36]. Others advocate for more life-like personas with carefully considered details [37,38]. Some details added to a persona may make the fictitious person more tangible, but some argue that these details could be irrelevant, extraneous, or distracting, potentially even introducing unnecessary bias. In this study, we used a more complete level of detail, incorporating demographic characteristics as well as opinions related to the topic at hand. The aim was to provide enough context to support empathy, design decisions, and communication strategies when access to user interviews was minimal. Future work should explore the impact of different levels of detail in personas on the tool’s effectiveness in public health informatics projects.

While evaluating the personas and their use within live public health informatics projects was beyond the scope of this study, evaluation is an important step. Future work should include pilot testing with designers, developers, and public health practitioners to assess the personas’ usefulness and their impact.

Additionally, more can be done to ensure the inclusiveness of personas. This can be accomplished with further user inquiry focusing on historically marginalized groups. Persona Cards could also be iteratively reviewed with groups of people whom they presume to represent to check for validity and identify any constructed biases. Without additional resources to conduct focus groups or user research, other processes can be implemented to better represent these communities, such as a backwards user journey [30].

### Conclusions

This study describes the process of developing personas from existing survey datasets. The mixed methods approach, combining quantitative cluster analysis with qualitative data integration, resulted in 13 detailed personas. These personas may be helpful in various stages of public health technology initiatives, including needs assessment, design, roll-out, and evaluation. By bridging the gap between large-scale data and actionable insights, these personas offer a powerful tool for creating more user-centered and effective public health information systems. The methodology presented here provides a flexible framework that can be adapted to meet the specific needs of various public health initiatives, potentially enhancing the use of UCD principles and thus the user-centeredness and effectiveness of public health technologies.

## Appendix

Multimedia Appendix 1 Statewide survey instrument. The complete 32-question survey instrument used for statewide data collection.

Multimedia Appendix 2 Second phase survey instrument. The complete 53-question survey instrument used for the second phase of data collection.

Multimedia Appendix 3 Additional descriptives.

Multimedia Appendix 4 Persona cards. The complete set of 13 persona cards developed through this research.

## Abbreviations

UCD: user-centered design
UW: University of Washington
