# Supplementary material for: Persona Development in Washington State: Mixed Methods Approach Using Statewide Survey Data
Source: Online J Public Health Inform. 2026 Mar 31;18:e75422. doi: 10.2196/75422 (PMC13080291; doi:10.2196/75422)
Supplement: Multimedia Appendix 2 [file ojphi_v18i1e75422_app2.pdf]

# Public Health Technology in Washington Follow-up Survey

The goal of this follow-up survey is to update information collected in 2022. To thank you for your time, you will receive a $10 Amazon gift card by email within two weeks of completing the follow-up survey. At the end of this survey, you will be asked if you would like to participate in an additional telephone or Zoom interview to talk more about your experiences with and share your thoughts on technology and public health. Compensation for the additional interview will be a $50 Amazon gift card.

If you have questions about this survey or want to speak with someone before agreeing to participate, please contact our team at waverify@uw.edu.

---

I am at least 18 years old; I live in Washington State; and I agree to participate in this survey.

Are all of the above true for you?

◯ Yes
◯ No

**We want to be sure we are reaching a wide range of Washington residents with this survey. Please tell us a little about yourself.**

| | |
|---|---|
| Which county do you live in? | ◯ Asotin<br>◯ Benton<br>◯ Chelan<br>◯ Clallam<br>◯ Clark<br>◯ Columbia<br>◯ Cowlitz<br>◯ Douglas<br>◯ Ferry<br>◯ Franklin<br>◯ Garfield<br>◯ Grant<br>◯ Grays Harbor<br>◯ Island<br>◯ Jefferson<br>◯ King<br>◯ Kitsap<br>◯ Kittitas<br>◯ Klickitat<br>◯ Lewis<br>◯ Lincoln<br>◯ Mason<br>◯ Okanogan<br>◯ Pacific<br>◯ Pend Oreille<br>◯ Pierce<br>◯ San Juan<br>◯ Skagit<br>◯ Skamania<br>◯ Snohomish<br>◯ Spokane<br>◯ Stevens<br>◯ Thurston<br>◯ Wahkiakum<br>◯ Walla Walla<br>◯ Whatcom<br>◯ Whitman<br>◯ Yakima |
| How old are you? | ◯ 18-29<br>◯ 30-39<br>◯ 40-49<br>◯ 50-59<br>◯ 60-69<br>◯ 70-79<br>◯ 80+ |
| Are you a parent or guardian to a child under 18 years who lives with you? | ◯ Yes<br>◯ No |
| What is the highest level of education you have completed? | ◯ Less than high school<br>◯ High school graduate or GED completion<br>◯ 2 year degree or some college<br>◯ 4 year degree or more |

| | |
|---|---|
| How do you describe yourself? | ◯ Female<br>◯ Male<br>◯ Transgender<br>◯ Non-binary/non-conforming<br>◯ Prefer not to respond |
| How would you describe your race and ethnicity? (Choose all that apply) | ☐ American Indian-Alaskan Native (AIAN)<br>☐ Asian<br>☐ Black<br>☐ Hispanic/Latinx<br>☐ Native Hawaiian and Other Pacific Islander (NHOPI)<br>☐ White<br>☐ Other |
| Do you speak a language other than English at home? | ◯ Yes<br>◯ No |
| If yes, what is that language? | __________________________________ |
| Have you traveled in the last 6 months or are you planning on traveling in the next 6 months? | ◯ Yes<br>◯ No |
| Please indicate the type of travel you completed and/or are planning (check all that apply): | ☐ Domestic<br>☐ International |
| Have you had any COVID-19 vaccines? | ◯ Yes<br>◯ No<br>◯ Prefer not to respond |

**Next, we'd like to ask you about your access to and experiences with technology. Here, we are interested in your general experiences, not your input on a specific app, tool or system.**

**How much do you agree or disagree with each of the following statements? Please select one response for each statement.**

| | Strongly Agree | Somewhat Agree | Neutral | Somewhat Disagree | Strongly Disagree |
|---|---|---|---|---|---|
| I am very confident using computers, smartphones, and other devices to access the Internet | ○ | ○ | ○ | ○ | ○ |
| In general, I trust information I find on the Internet | ○ | ○ | ○ | ○ | ○ |
| When I get a new electronic device, or need to do a new task on it, I usually need someone else to set it up or help me. | ○ | ○ | ○ | ○ | ○ |
| I have a hard time learning how to use new technology, devices and software programs | ○ | ○ | ○ | ○ | ○ |
| Technology gives me more control over my daily life | ○ | ○ | ○ | ○ | ○ |
| I worry about privacy and am concerned that information I send over the Internet will be seen by other people | ○ | ○ | ○ | ○ | ○ |
| I worry about being able to afford new computing devices as technology changes and improves | ○ | ○ | ○ | ○ | ○ |
| I am very confident that I can securely do business online (for example: shopping, banking, using social media, using my health care organization's patient account, etc.) | ○ | ○ | ○ | ○ | ○ |

Which of the following are concerns you have when it comes to accessing and using the Internet:

Please check all that apply

- ☐ How my data and information is being used (including ways that I may not be aware of)
- ☐ Ensuring the safety and security of my personal information (such as banking or health information)
- ☐ Protecting myself from online criminal activities (cyberstalking, hacking, cyberbullying)
- ☐ Protecting my computer from online viruses and malware
- ☐ None of these concern me

| | |
|---|---|
| What impact do you believe the Internet and technology have on society?<br><br>Please check one | ◯ Totally Beneficial or Positive<br>◯ Mostly Beneficial or Positive<br>◯ Both Beneficial and Harmful<br>◯ Mostly Harmful or Negative<br>◯ Totally Harmful or Negative |
| Do you have a smartphone? | ◯ Yes<br>◯ No<br>(A smartphone is a mobile phone that can connect to the Internet and run apps.) |

## Now, we'd like to ask you some questions about tools provided by public health agencies.

The Washington Department of Health provides a smartphone-based COVID-19 vaccination verification tool called WA Verify.

WA Verify is designed to make it easier for vaccinated individuals to keep track of and provide vaccine verification on the go, right from their smart phones.

How it Works. After confirming it is you using your date of birth, email address or phone number, the WA Verify system looks for your COVID-19 vaccination records in the state database. It sends a link via text or email that only you can access (because you have created a PIN). When you access the link, you can download a QR code and human-readable version of your COVID-19 immunization records from your smartphone. This QR code can be shown as you enter an event or venue to verify your vaccine status.

**Thinking about a public health tool like WA Verify, how much do you agree or disagree with each of the following statements? Please select one response for each statement**

| | Strongly Agree | Somewhat Agree | Neutral | Somewhat Disagree | Strongly Disagree |
|---|---|---|---|---|---|
| I am very confident that information I share with public health is secure | ◯ | ◯ | ◯ | ◯ | ◯ |
| In general, I trust information from public health | ◯ | ◯ | ◯ | ◯ | ◯ |
| In general, I can trust tools developed by public health | ◯ | ◯ | ◯ | ◯ | ◯ |
| With public health systems and tools, I worry about privacy and am concerned that information I send will be seen by other people | ◯ | ◯ | ◯ | ◯ | ◯ |
| I am very confident that a public health tool like WA Verify is secure | ◯ | ◯ | ◯ | ◯ | ◯ |
| I trust that public health will use my information to help keep my community healthy | ◯ | ◯ | ◯ | ◯ | ◯ |
| I am concerned that public health tools will be used to track me | ◯ | ◯ | ◯ | ◯ | ◯ |
| I trust that public health tools like WA Verify are safe from hacking | ◯ | ◯ | ◯ | ◯ | ◯ |

What impact do you believe public health tools like WA Verify have or could have on society?

Please check one

◯ Totally Beneficial or Positive
◯ Mostly Beneficial or Positive
◯ Both Beneficial and Harmful
◯ Mostly Harmful or Negative
◯ Totally Harmful or Negative

Please elaborate on your answer: ____________________________

**Some businesses, organizations and venues have asked that a COVID-19 vaccination card or a negative COVID-19 test result is presented before allowing people to enter their building or use their services. In this section, we would like to learn about your experiences with showing proof of COVID-19 vaccination or COVID-19 test results. When answering these questions, please try to think back to when more restrictions were in place, around mid-2021.**

| | |
|---|---|
| Have you ever been asked to show proof of COVID-19 vaccination before participating in an activity or entering a business? (For example, going to a healthcare facility, traveling, attending a religious service or social event etc.) | ◯ Yes<br>◯ No |
| When you were asked to show proof of vaccination, did you use WA Verify to verify your vaccine status? | ◯ Yes<br>◯ No<br>◯ Not sure |
| How was the process of verifying your vaccine status using WA Verify?<br><br>Check all that apply | ☐ The process was easy<br>☐ I showed WA Verify, but it took more time than showing my paper vaccine card<br>☐ I showed WA Verify but I also had to show my paper vaccine card<br>☐ I had trouble with the process<br>☐ I showed WA Verify, but they QR code was not scanned, they just let me in<br>☐ Other |
| Please include any additional comments you have on the process of using WA Verify. | __________________________________ |
| As a part of our evaluation project we will be talking to a sample of businesses and organizations about their vaccine verification process. We would appreciate you listing one or two of the businesses, events or venues where you were asked to provide proof of vaccination status. Again, you may need to think back to 2021.<br><br>Your name and information about you will not be shared when we contact these businesses and organizations. | __________________________________ |
| Have you ever been asked to show proof of negative COVID-19 test before participating in an activity or entering a business? (For example, going to a healthcare facility, traveling, attending a religious service or social event etc.) | ◯ Yes<br>◯ No |

**In this section we will ask questions to help us understand more about why people choose to use or not to use a portable electronic COVID-19 vaccine record like WA Verify.**

| | |
|---|---|
| Do you have or have you ever had a WA Verify QR code on your phone? | ◯ Yes<br>◯ No<br>◯ Not sure |

**Below is a list of opinions about using a tool like WA Verify. Please indicate your agreement or disagreement with these statements.**

| | Strongly Agree | Somewhat Agree | Neutral | Somewhat Disagree | Strongly Disagree |
|---|---|---|---|---|---|
| I have no need to show my vaccination record | ○ | ○ | ○ | ○ | ○ |
| Having my vaccination information  on my phone is convenient | ○ | ○ | ○ | ○ | ○ |
| The benefits of using a tool like this outweigh any risks | ○ | ○ | ○ | ○ | ○ |
| I'm concerned about data security when it comes to personal health data like this | ○ | ○ | ○ | ○ | ○ |
| I'd rather not carry a paper COVID-19 vaccine card | ○ | ○ | ○ | ○ | ○ |
| The risks associated with using a tool like this outweigh any benefits to myself, my family or my community | ○ | ○ | ○ | ○ | ○ |

**The Department of Health is considering ways public health tools can help keep communities safe, maintain secure access to important health information, and support exchange of information in an emergency. Below are some examples of tools that public health might support. Please indicate how you would prioritize these tools.**

| | High Priority | Medium Priority | Low Priority | Not a Priority | Not Sure |
|---|---|---|---|---|---|
| A tool to display COVID-19 immunization status. Example: A person can show a QR code, stored on their phone, to verify immunization status. | ○ | ○ | ○ | ○ | ○ |
| A tool that maintains up to date COVID-19 immunization records for family members. Example: A parent, guardian or caregiver could provide a QR code, stored on their phone, to verify COVID immunization status for a person in their care. | ○ | ○ | ○ | ○ | ○ |
| A tool that displays a full immunization record. (COVID and others such as influenza, Tdap, Varicella, etc.) Example: An employee could prove they have the necessary vaccinations by providing a QR code to their employer. A parent could provide a QR code, stored on their phone, to verify immunization status for a child's school or childcare center. | ○ | ○ | ○ | ○ | ○ |
| A tool providing access to advance directives. Example: An individual or their spouse, could show a QR code on their phone to allow healthcare providers to quickly access their previously documented advance directives about end-of-life medical care. | ○ | ○ | ○ | ○ | ○ |

| | | | | | |
|---|---|---|---|---|---|
| A tool to share access to lab results for notifiable conditions. Example: If a lab test is conducted for a reportable condition, that result could be provided to providers or public health professionals through a QR code stored on a person's phone. | ○ | ○ | ○ | ○ | ○ |
| A tool to facilitate access to medical records while traveling. Example:  Individuals would be able to provide a summary of their medical records to providers while traveling by using a QR code stored on their phone. At a minimum, the record would include medications, allergies, and the problem list. | ○ | ○ | ○ | ○ | ○ |

---

Thank you very much for your time. Is there anything else you would like to share with us about your thoughts and experience related to public health tools and technologies? ______________________________________

**The WA Verify Evaluation team is conducting 30-minute interviews to hear more about your thoughts and concerns about WA Verify and other public health tools. Those who complete the 30-minute Zoom or telephone interview will receive a $50 Amazon gift card.**

| | |
|---|---|
| Are you interested in being contacted to schedule an interview? | ◯ Yes<br>◯ No |
| The email we have on file for you is [email], is this the address you would like us to use? | ◯ Yes<br>◯ No |
| Please enter a better email address: | ________________________ |
