# Supplementary material for: Persona Development in Washington State: Mixed Methods Approach Using Statewide Survey Data
Source: Online J Public Health Inform. 2026 Mar 31;18:e75422. doi: 10.2196/75422 (PMC13080291; doi:10.2196/75422)
Supplement: Multimedia Appendix 3 [file ojphi_v18i1e75422_app3.docx]

Supplemental Descriptive Tables for Persona Development in Washington State: Mixed-Methods Approach Using Statewide Survey Data

While the personas development analysis did not utilize all questions asked on the follow-up survey, we present some additional descriptive statistics based on these questions to provide some context. Follow-up survey respondents were asked questions regarding their trust in public health, reasons to use or not use WA Verify and future potential uses of WA Verify. The questions and responses are summarized in tables in the following pages. Each row corresponds to a question, and each column corresponds to a possible response. Each other cell denotes the count of individuals responding with the given response to the given question as well as the row percentage provided in parentheses.

## Trust in Public Health

The survey asked the following question: “Thinking about a public health tool like WA Verify, how much do you agree or disagree with each of the following statements? Please select one response for each statement:”

|  | **Strongly Agree** | **Somewhat Agree** | **Neutral** | **Somewhat Disagree** | **Strongly Disagree** | **Missing** |
| --- | --- | --- | --- | --- | --- | --- |
| I am very confident that information I share with public health is secure | 29 (20.3%) | 70 (49%) | 22 (15.4%) | 17 (11.9%) | 4 (2.8%) | 1 (0.7%) |
| In general, I trust information from public health | 55 (38.5%) | 57 (39.9%) | 22 (15.4%) | 5 (3.5%) | 3 (2.1%) | 1 (0.7%) |
| In general, I can trust tools developed by public health | 43 (30.1%) | 61 (42.7%) | 27 (18.9%) | 7 (4.9%) | 4 (2.8%) | 1 (0.7%) |
| I worry about privacy and am concerned that information I send will be seen by other people | 15 (10.5%) | 41 (28.7%) | 32 (22.4%) | 42 (29.4%) | 12 (8.4%) | 1 (0.7%) |
| I am very confident that a public health tool like WA Verify is secure | 23 (16.1%) | 71 (49.7%) | 29 (20.3%) | 15 (10.5%) | 3 (2.1%) | 2 (1.4%) |
| I trust that public health will use my information to help keep my community healthy | 54 (37.8%) | 53 (37.1%) | 27 (18.9%) | 6 (4.2%) | 2 (1.4%) | 1 (0.7%) |
| I am concerned that public health tools will be used to track me | 10 (7%) | 36 (25.2%) | 25 (17.5%) | 38 (26.6%) | 33 (23.1%) | 1 (0.7%) |
| I trust that public health tools like WA Verify are safe from hacking | 8 (5.6%) | 45 (31.5%) | 46 (32.2%) | 29 (20.3%) | 12 (8.4%) | 3 (2.1%) |

## Reasons to Use or Not use WA Verify

The survey asked the following question: “Below is a list of opinions about using a tool like WA Verify. Please indicate your agreement or disagreement with these statements:”

|  | **Strongly Agree** | **Somewhat Agree** | **Neutral** | **Somewhat Disagree** | **Strongly Disagree** | **Missing** |
| --- | --- | --- | --- | --- | --- | --- |
| I have no need to show my vaccination record | 17 (11.9%) | 27 (18.9%) | 37 (25.9%) | 30 (21%) | 29 (20.3%) | 3 (2.1%) |
| Having my vaccination information on my phone is convenient | 78 (54.5%) | 42 (29.4%) | 14 (9.8%) | 0 (0%) | 6 (4.2%) | 3 (2.1%) |
| The benefits of using a tool like this outweigh any risks | 40 (28%) | 47 (32.9%) | 37 (25.9%) | 9 (6.3%) | 7 (4.9%) | 3 (2.1%) |
| I'm concerned about data security when it comes to personal health data like this | 12 (8.4%) | 47 (32.9%) | 40 (28%) | 29 (20.3%) | 12 (8.4%) | 3 (2.1%) |
| I'd rather not carry a paper COVID-19 vaccine card | 60 (42%) | 20 (14%) | 39 (27.3%) | 10 (7%) | 11 (7.7%) | 3 (2.1%) |
| The risks associated with using a tool like this outweigh any benefits to myself, my family or my community | 13 (9.1%) | 22 (15.4%) | 43 (30.1%) | 26 (18.2%) | 36 (25.2%) | 3 (2.1%) |

## Future Uses of WA Verify

The survey asked the following question: “The Department of Health is considering ways public health tools can help keep communities safe, maintain secure access to important health information, and support exchange of information in an emergency. Below are some examples of tools that public health might support. Please indicate how you would prioritize these tools”. Note that individuals could rate multiple options as high (or any) priority. Each cell provides the count of responses as well as row percentages.

|  | **High Priority** | **Medium Priority** | **Low Priority** | **Not A Priority** | **Not Sure** | **Missing** |
| --- | --- | --- | --- | --- | --- | --- |
| A tool to display COVID-19 immunization status. Example: A person can show a QR code, stored on their phone, to verify immunization status. | 44 (30.8%) | 46 (32.2%) | 27 (18.9%) | 13 (9.1%) | 8 (5.6%) | 5 (3.5%) |
| A tool that maintains up to date COVID-19 immunization records for family members. Example: A parent, guardian or caregiver could provide a QR code, stored on their phone, to verify COVID immunization status for a person in their care. | 31 (21.7%) | 66 (46.2%) | 22 (15.4%) | 14 (9.8%) | 5 (3.5%) | 5 (3.5%) |
| A tool that displays a full immunization record. (COVID and others such as influenza, Tdap, Varicella, etc.) Example: An employee could prove they have the necessary vaccinations by providing a QR code to their employer. A parent could provide a QR code, stored on their phone, to verify immunization status for a child's school or childcare center. | 58 (40.6%) | 46 (32.2%) | 19 (13.3%) | 11 (7.7%) | 4 (2.8%) | 5 (3.5%) |
| A tool providing access to advance directives. Example: An individual or their spouse, could show a QR code on their phone to allow healthcare providers to quickly access their previously documented advance directives about end-of-life medical care. | 59 (41.3%) | 46 (32.2%) | 17 (11.9%) | 11 (7.7%) | 6 (4.2%) | 4 (2.8%) |
| A tool to share access to lab results for notifiable conditions. Example: If a lab test is conducted for a reportable condition, that result could be provided to providers or public health professionals through a QR code stored on a person's phone. | 43 (30.1%) | 50 (35%) | 31 (21.7%) | 11 (7.7%) | 3 (2.1%) | 5 (3.5%) |
| A tool to facilitate access to medical records while traveling. Example: Individuals would be able to provide a summary of their medical records to providers while traveling by using a QR code stored on their phone. At a minimum, the record would include medications, allergies, and the problem list. | 65 (45.5%) | 43 (30.1%) | 19 (13.3%) | 4 (2.8%) | 7 (4.9%) | 5 (3.5%) |
