# Supplementary material for: Persona Development in Washington State: Mixed Methods Approach Using Statewide Survey Data
Source: Online J Public Health Inform. 2026 Mar 31;18:e75422. doi: 10.2196/75422 (PMC13080291; doi:10.2196/75422)
Supplement: Multimedia Appendix 4 [file ojphi_v18i1e75422_app4.pdf]

David

Views technology as a pathway to progress

Occupation: Software Engineer

Age: 43

Race/Ethnicity: White

English Only Home: Yes

Region: Western WA

Education: 4-year

Has Minor Child: Yes

Gender: Male

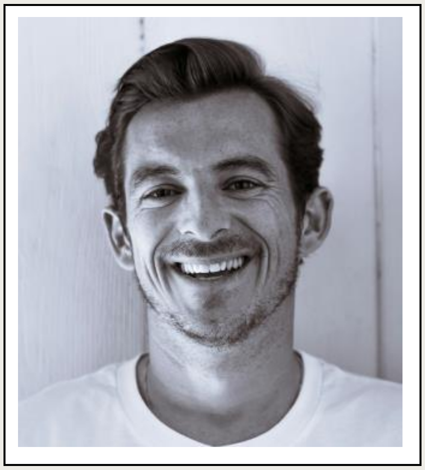

Public Health & Tech Characteristics

- High Level of Support for PH Policies
- Owns a Smartphone
- High Tech Readiness
- Uses Online Health Tools

Drivers

- Advocates for the use of technology
- Values data and having access to data, despite known risks
- Believes data can be beneficial for solving society's problems
- Views technology as a way to support his autonomy and feeling in control of his life

Quotes

"Let's use the newest and best technology available to help address community issues including public health issues."

"I am of the opinion that more access to more data is good. Some might not like their data being shared, but I believe that individuals must assume some risk, hopefully very slight, to aid the greater good."

"I appreciate being able to easily access health information about my parents and my kids."

"One of the many challenges that come with being a divorced parent has been record-keeping and scheduling. Can we keep health information online rather than on paper so my co-parent and I can both have easy? This would help so much in signing up for camps and other activities that require immunizations or physicals."

Yuki

Enthusiastic yet cautious user of health technology

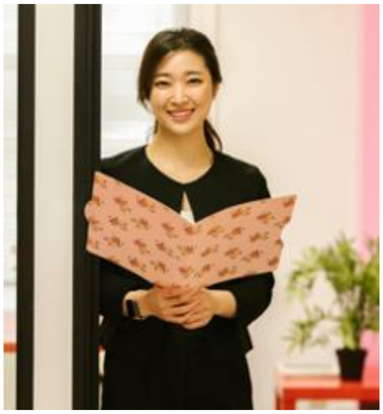

Occupation: PT Assistant

Age: 40

Race/Ethnicity: Asian

English Only Home: No

Region: Western WA

Education: 4-year

Has Minor Child: Yes

Gender: Female

Public Health & Tech Characteristics

High Level of Support for PH Policies

Owns a Smartphone

High Tech Readiness

Uses Online Health Tools

Drivers

- Needs reassurance of benefits as well as how well risks have been handled before fully trusting a new digital tool
- Wants to feel very confident in a system before advocating for its use by her parents
- Appreciates the convenience of personal health tools

Quotes

"Convenience is a big deal when it comes to health tools. It can be a game-changer for keeping us informed and safe – both personally and as a community."

"I appreciate when health tools have translations so that my parents, who speak Japanese, can easily use them."

"Convenience is key for me, and I find online tools helpful for tracking my and the kids' health records."

"I value having easy access to my parents' medical records in Japanese."

Emily

Concerned about social justice and inequities

Occupation: Social Services

Region: Western WA

Age: 32

Education: 4-year

Race/Ethnicity: White

Has Minor Child: No

English Only Home: Yes

Gender: Female

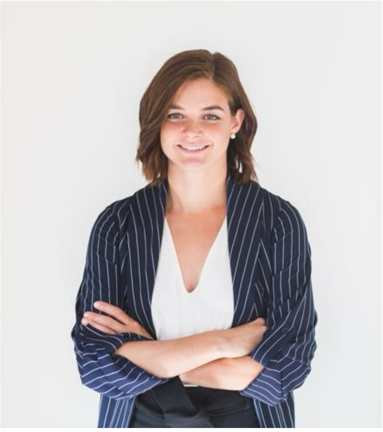

Public Health & Tech Characteristics

- High Level of Support for PH Policies
- Owns a Smartphone
- High Tech Readiness
- Uses Online Health Tools

Drivers

- Passionate about inclusivity, including in public health
- Concerned about digital tools that might exclude or exploit vulnerable populations
- Values the potential of public health measures but wary of government abuse
- Believes in access and information but also autonomy

Quotes

“Especially in the cases of marginalized communities who don't trust these kind of health mandates due to systemic issues, this could be a way to further alienate them. You have to consider the historical context and build trust through transparent efforts.”

“The politicization of health tools is so polarizing. It's diminished the beneficial opportunities these tools could have had. Even though I want to partake in public health and do what I can, the social context and the clear intent to track medical decisions, like abortion care, make me somewhat distrustful of these programs.”

Ryan

Tracking-wary and tech avoidant

Occupation: Grocery Stocker

Age: 52

Race/Ethnicity: White

English Only Home: Yes

Region: Western WA

Education: 4-year

Has Minor Child: No

Gender: Male

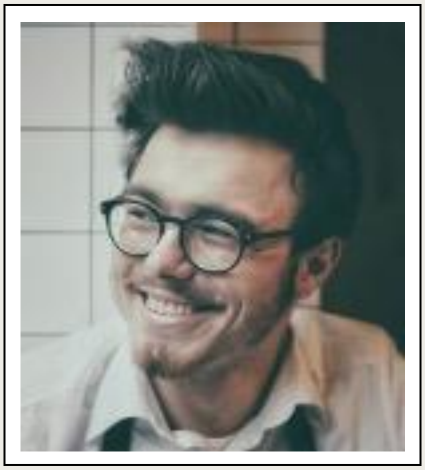

Public Health & Tech Characteristics

- High Level of Support for PH Policies
- Owns a Smartphone
- High Tech Readiness
- Uses Online Health Tools

Drivers

- Strong sense of individuality and non-conformity
- Worries about government tracking, values privacy
- Skeptical about the benefits of technology outweighing its risks
- Needs to know tools are efficient, non-intrusive, and transparent before considering their adoption

Quotes

"Public health is crucial, no doubt. But sometimes, the mainstream solutions feel a bit one-size-fits-all. I'm more into personalized approaches, you know?"

"I don't want to be tracked."

"I think we all need to keep our eyes open to the ways we give over control and resist that. The complacency and blind trust will be our downfall if we're not careful."

Desmond

Technically savvy and embraces the convenience technology offers

Occupation: Banking

Age: 37

Race/Ethnicity: Black

English Only Home: Yes

Region: Western WA

Education: 4-year

Has Minor Child: No

Gender: Male

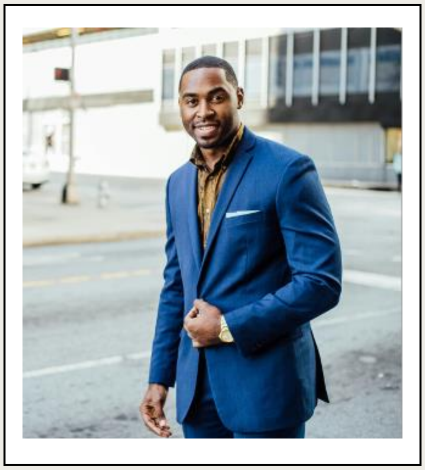

Public Health & Tech Characteristics

- High Level of Support for PH Policies
- Owns a Smartphone
- High Tech Readiness
- Uses Online Health Tools

Drivers

- Believes technology makes everything convenient but is wary of sharing data online
- Has witnessed malicious use of stolen information through his work
- Values personal safety and uses tools that will support his own and his family's safety
- Aware of the danger of algorithms perpetuating bias

Quotes

"Data and technology can be so helpful and really help make things faster and more convenient in everyday life. The problem is that there are aways people with malicious intent out there who can use any information they get their hands on to cause harm."

"I only have one dedicated credit card that I use online and I track it regularly. I am hesitant to create new accounts and share health or other personal information online. I weigh the potential risks with the benefits to make a decision about any new app or account."

"I am often out and about and since my wife has an autoimmune disease, I really value the security of knowing that there is some level of safeguard around us. To us, downloading and using online public health tools are usually worth it."

Maria

Focused on the happiness and health of her close family

Occupation: Human Resources

Age: 35

Race/Ethnicity: Hispanic

English Only Home: Yes

Region: Eastern WA

Education: ≤ 2-year

Has Minor Child: No

Gender: Female

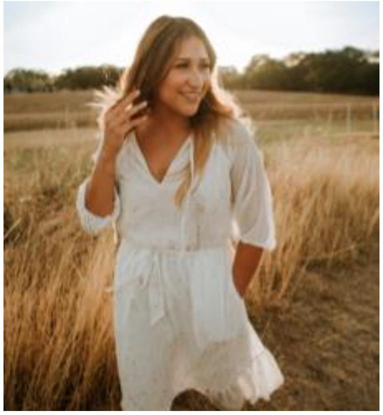

Public Health & Tech Characteristics

- High Level of Support for PH Policies
- Owns a Smartphone
- High Tech Readiness
- Uses Online Health Tools

Drivers

- Driven by her commitment to her family, prioritizes their health and safety
- Feels a responsibility to stay up to date with the latest information to keep her loved ones safe
- Hesitant about adopting new technologies because of potential risks and uncertainties

Quotes

“In all honesty, I don't feel fully in the loop about the whole public health scene. But I make it a point to share any news about recalls or outbreaks with my family. It's my way of contributing to their safety.”

“At work I use the tools I'm required to use to check on flu and COVID vacciations and it does make things easier having the information electronically.”

“I don't like the idea of other people knowing my health status or information. I don't really have anything to hide, but I get why others wouldn't want everyone to know that. I don't think these type of things should be required. People have reasons for worrying.”

Linda

Not a fan of the smartphone, appreciates snail mail and hard copies

Occupation: Retired

Age: 66

Race/Ethnicity: White

English Only Home: Yes

Region: Western WA

Education: ≤ 2-year

Has Minor Child: No

Gender: Female

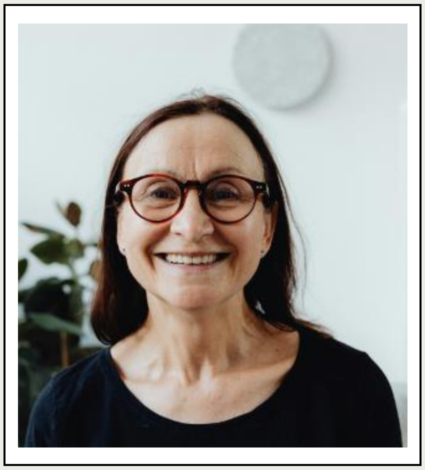

Public Health & Tech Characteristics

- High Level of Support for PH Policies
- Owns a Smartphone
- Lower Tech Readiness
- Uses Online Health Tools

Drivers

- Concerned about being left behind as technology advances
- Values her independent and active lifestyle
- Prefers to use lower tech ways of interacting with important information (physical copies, mailings, etc.)

Quotes

"I don't like my iPhone, don't always want to or remember to bring it with me. Plus, I already have too many apps and it makes it hard for me to navigate on my phone because my hands shake and my eyes are getting worse."

"I like having things down on paper and receiving important documents and information by mail. My vaccine card doesn't need to be charged."

Richard

Concerned about financial barriers to newer technology

Occupation: Appliance Repair

Age: 60

Race/Ethnicity: White

English Only Home: Yes

Region: Western WA

Education: ≤ 2-year

Has Minor Child: No

Gender: Male

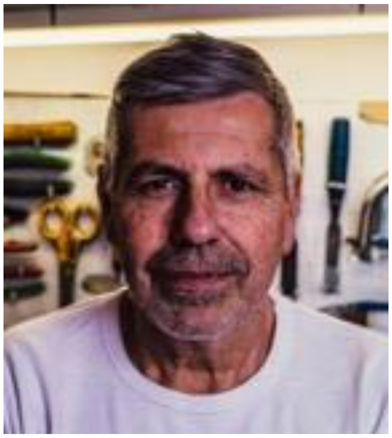

Public Health & Tech Characteristics

- High Level of Support for PH Policies
- Owns a Smartphone
- Lower Tech Readiness
- Uses Online Health Tools

Drivers

- Appreciates simplicity in life and technology
- Needs a very strong reason to adopt a new tool or try a new technology
- Focuses on spending his time doing the things he enjoys, not spending time on new tools

Quotes

“You have to understand, I've had a lot of very frustrating experiences with technology. I had a lot of trouble getting my doctor's app to work on my phone--she wanted me to set it up to send messages.”

“I think some apps just won't work because my phone is too old (for example, I don't think I have a digital wallet on my phone).”

“I got this phone four years ago but it still makes calls and texts so that works for me, plus the brand new phones are just not in my budget.”

“I would consider using a new app from public health if my doctor recommended it. If it was free, simple, and there was a great reason for me to spend the time to figure out how it works and get it up and running on my phone.”

“I might need to get someone to help set [an app] up. I have a neighbor who sometimes helps me with computer questions but I don't want to bother her if it isn't real important.”

Patricia

Retired paralegal, loves her church community and service activities

Occupation: Retired

Age: 67

Race/Ethnicity: White

English Only Home: Yes

Region: Western WA

Education: 4-year

Has Minor Child: No

Gender: Female

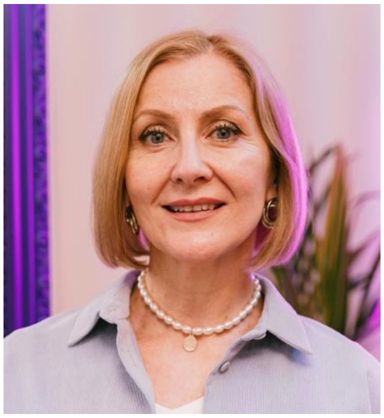

Public Health & Tech Characteristics

- High Level of Support for PH Policies
- Owns a Smartphone
- High Tech Readiness
- Uses Online Health Tools

Drivers

- Does not want to be forced to spend more time on devices
- Concerned for marginalized communities and unequal access to technology
- Appreciates what apps and technology can do to promote health

Quotes

"I like the idea of using technology to help keep people healthy but I do worry about the marginalized communities with whom I spend time through my church service activities. How would technology based interventions affect them or possibly exclude them? At the same time, protecting the public from infection is good for everyone, kind of like the whole 'herd immunity' idea."

"I personally have no problem using an app and would like to have my health info on my phone. I'm not sure about my husband though. If I could have an app for him too, that could be a solution."

"We both feel more comfortable when we have a paper copy of things. I always print out the directions when I'm going somwhere new, in case my phone dies!"

"An app would need to be easy to use and easy to find on my phone."

Kyle

Fierce defender of his family and his autonomy

Occupation: Firefighter

Age: 31

Race/Ethnicity: White

English Only Home: Yes

Region: Eastern WA

Education: 4-year

Has Minor Child: Yes

Gender: Male

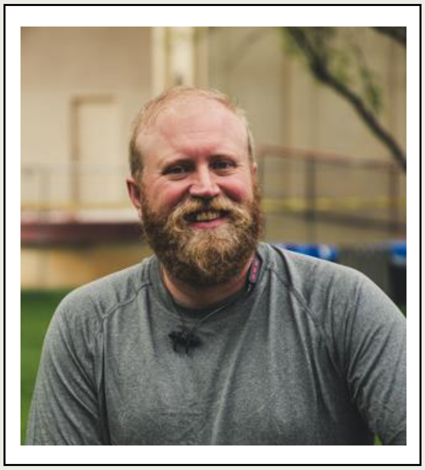

Public Health & Tech Characteristics

- Low Level of Support for PH Policies
- Owns a Smartphone
- High Tech Readiness
- Does not use Online Health Tools

Drivers

- Personal agency is paramount, particularly around health decisions
- Master of his own fate
- Distrust of big government and big pharma
- Passionate advocate for and protector of family

Quotes

"I will not allow my data or my families data to become the property of the US government. I cannot imagine any scenario where I would knowingly use a government-supported tool so that they can track us."

"Government public health tools are now political tools, whether we want them to be or not."

"I don't trust that my health information wouldn't be used in some way by the government, or sold to industry for targeted marketing or discriminatory practices towards me or others."

"My family is very healthy. We take our vitamins, drink water, eat healthy, and exercise. People need to get off the couch."

"Healthy individuals should have control of their own health decisions, not be forced to do something because of those who are unhealthy."

Alicia

Tech savvy but protective when it comes to her family

Occupation: Teacher

Age: 46

Race/Ethnicity: Black

English Only Home: Yes

Region: Western WA

Education: 4-year

Has Minor Child: Yes

Gender: Female

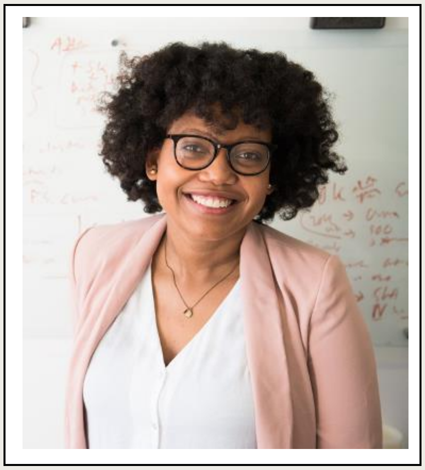

Public Health & Tech Characteristics

High Level of Support for PH Policies

Owns a Smartphone

High Tech Readiness

Uses Online Health Tools

Drivers

- Trusts her community for health and parenting information
- Willing to take on some risk for the greater good when it comes to herself but not willing to have her daughter take those risks
- Considers a healthy work and family balance when making decisions around technology

Quotes

"I will get vaccines because of my age but I won't put my daughter's health at risk. The COVID vaccine, for example is needed for people with underlying conditions, but I don't want to risk my daughter's future and ability to have children just to check a box. All of the kids are still getting COVID, vaccinated or not!"

"I have enough to do without another task of keeping some app up-to-date."

"If I'm going to use another app there needs to be a real value in it for me. Is it going to save me time, money or annoyance? Can it help me get my daughter's physical information to her school more quickly for sports registration?"

"If someone at church has a good experience with an online health tool, I might consider trying it."

Isabel

Passionate about technology but has reservations regarding privacy

Occupation: Pre-med Student

Age: 21

Race/Ethnicity: Hispanic

English Only Home: No

Region: Western WA

Education: ≤ 2-year

Has Minor Child: No

Gender: Female

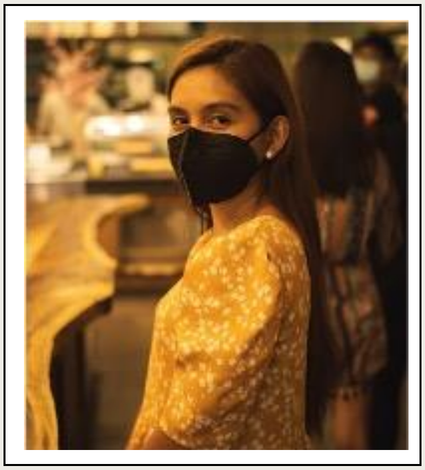

Public Health & Tech Characteristics

- High Level of Support for PH Policies
- Owns a Smartphone
- High Tech Readiness
- Uses Online Health Tools

Drivers

- Needs to know all of the considerations and risks before making a decision about using a new technology
- Hopeful about the potential of public health tools but feels there is often too much personal risk for some with data privacy and any unintended consequences
- Driven to advocate for vulnerable populations such as undcoumented individuals

Quotes

“In theory, I love the idea of keeping track of important health information online through the state public health authority because I do trust public health. However, I have a deep learned cautiousness about sharing information with governement entities and authorities because my parents are undocumented.”

“I weigh the benefits and risks of sharing information online and don't take the process of entering my information online, financial or otherwise, lightly. I am one of those people who does read the fine print. I want to have details on how my data will be collected and used.”

“I know that apps for health and public health could really help some people, but I also think about some of my extended family who live in the US but are not completely comfortable speaking English. Any tool should be accessible to those who aren't proficient in Engish.”

“I feel that any tool like this should be a choice and should not be mandated, we should have options to accomplish the same thing without using an app.”

Riley

Grad student looking at the big picture

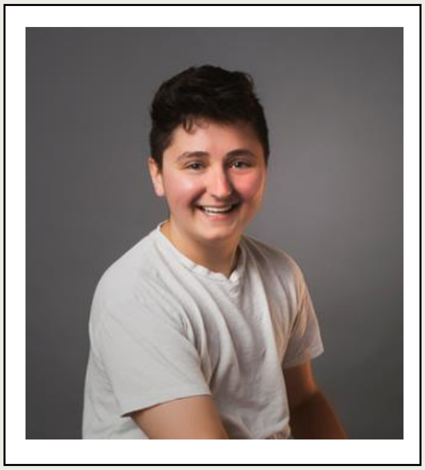

Occupation: Grad Student

Age: 26

Race/Ethnicity: White

English Only Home: Yes

Region: Western WA

Education: 4-year

Has Minor Child: No

Gender: Non-binary

Public Health & Tech Characteristics

- High Level of Support for PH Policies
- Owns a Smartphone
- High Tech Readiness
- Uses Online Health Tools

Drivers

- Frustrated by the slow pace of change and progress
- Driven by a desire to understand and address the root causes of injustices
- Values technology's ability to connect us but does not think it will solve all our problems

Quotes

“I really think we should be focussing on addressing the critical issues at hand. We have huge imminent threats like the climate crisis and we need universal access to free healthcare.”

“I'm just not convinced these tools we do anything. I think technology can be helpful in raising awareness and mobilizing people. But I just don't see what this is going to do.”

“… no matter what you do here half the population is going to demonize it or deny the existence of a problem.”
